# Unlocking the Potential: A Systematic Review of Master Protocol in Pediatrics

**DOI:** 10.1007/s43441-024-00656-z

**Published:** 2024-04-23

**Authors:** Yimei Li, Robert Nelson, Rima Izem, Kristine Broglio, Rajiv Mundayat, Margaret Gamalo, Yansong Wen, Haitao Pan, Hengrui Sun, Jingjing Ye

**Affiliations:** 1 Department of Biostatistics, Epidemiology and Informatics, University of Pennsylvania, 3501 Civic Center Blvd, Colket Translational Research Building Room 4032, 19034 Philadelphia, PA USA; 2 Department of Pediatrics, University of Pennsylvania, Philadelphia, PA USA; 3 Division of Oncology, The Children’s Hospital of Philadelphia, Philadelphia, PA USA; 4 Johnson & Johnson, Spring House, PA USA; 5 Statistical Methodology, Novartis Pharma AG, Basel, Switzerland; 6 AstraZeneca, Gaithersburg, MD USA; 7 Pfizer, New York, NY USA; 8 Department of Biostatistics, St. Jude Children’s Hospital, Memphis, TN USA; 9 Food & Drug Administration, Silver Spring, MD USA; 10 BeiGene, Cambridge, USA

**Keywords:** Master protocol, Pediatric, Systematic review, Platform trial, Basket trials, Umbrella trial

## Abstract

The use of master protocols allows for innovative approaches to clinical trial designs, potentially enabling new approaches to operations and analytics and creating value for patients and drug developers. Pediatric research has been conducted for many decades, but the use of novel designs such as master protocols in pediatric research is not well understood. This study aims to provide a systematic review on the utilization of master protocols in pediatric drug development. A search was performed in September 2022 using two data sources (PubMed and ClinicalTrials.gov) and included studies conducted in the past10 years. General study information was extracted such as study type, study status, therapeutic area, and clinical trial phase. Study characteristics that are specific to pediatric studies (such as age of the participants and pediatric drug dosing) and important study design elements (such as number of test drug arms and whether randomization and/or concurrent control was used) were also collected. Our results suggest that master protocol studies are being used in pediatrics, with platform and basket trials more common than umbrella trials. Most of this experience is in oncology and early phase studies. There is a rise in the use starting in 2020, largely in oncology and COVID-19 trials. However, adoption of master protocols in pediatric clinical research is still on a small scale and could be substantially expanded. Work is required to further understand the barriers in implementing pediatric master protocols, from setting up infrastructure to interpreting study findings.

**Supplementary Information:**

The online version contains supplementary material available at 10.1007/s43441-024-00656-z.

## Introduction

A master protocol is a novel clinical trial design that attempts to evaluate multiple experimental therapies in one or multiple indications, under one overarching protocol [1]. The definitions of a master protocol and its subtypes vary considerably in the medical literature [2]; however, it is reasonable to adopt the definitions proposed by the Food & Drug Administration (FDA) whereby there are three types of master protocols: umbrella, basket, and platform [3]. An umbrella trial evaluates multiple drugs or drug regimens for a single disease. A basket trial evaluates a single drug or drug regimen for multiple diseases. A platform trial studies multiple drugs for a single disease but allows drugs to enter or leave the platform based on a decision algorithm [1]. As such, a platform trial can be understood as an adaptive umbrella trial where substudies involving either treatment arms or study populations can be added or dropped during the trial [4, 5].

The use of a master protocol holds the promise of increasing efficiency and enabling new approaches to operations and analytics [6]. Pediatric drug development poses many challenges, some of which could be addressed by using master protocols. For example, sponsors are required to conduct pediatric studies (absent a waiver) for assets being developed for adult diseases. Thus multiple sponsors may need to conduct similar pediatric studies in the same, relatively small population. In this context, pediatric development requires prior agreement with health authorities regarding a plan that will result in pediatric labeling– a requirement that adds further complexity to achieving alignment if more than one sponsor is involved. Multiple sponsors enrolling pediatric patients into separate trials of drugs in the same disease or with the same mechanism of action is not efficient, while master protocols may be a good alternative design to improve efficiency [7]. For example, the NCI-COG Pediatric MATCH trial [8] aims to evaluate molecular-targeted therapies in biomarker-selected cohorts of pediatric patients with refractory cancers by screening tumors for actionable alterations. Because the diseases are rare, and the incidence of some genetic mutations targeted by a given therapy is low (e.g., less than 3% of patients with the disease), conducting a separate trial for each concurrently developed therapy will slow down recruitment and thus slow down access to potentially life-saving therapies for all patients. Conversely, a master protocol under a shared infrastructure/platform alleviates this issue by including many new therapies under one study so that more patients could receive appropriate treatment after molecular screening. In the NCI-COG Pediatric MATCH trial, actionable mutations were detected in 31.5% of the first 1000 tumors screened, and 13% of the screened patients were enrolled in a treatment arm [9].

Pediatric studies also pose ethical challenges, such as enrolling the minimum number of participants necessary to answer the scientific question [10, 11], and minimizing or avoiding exposure to placebo (e.g., some pediatric cancers, rare progressive genetic diseases, pediatric inflammatory bowel diseases) [12, 13]. In addition, there are many barriers to enrolling children in clinical trials, including technical limitations on blood draws and procedures, parental hesitance to permit their child to participate, and off-label prescribing of the investigational treatment outside of a clinical trial once it is approved in adults [14, 15]. With master protocols, more specifically platform trials, a shared concurrent control group is often used to compare with multiple experimental therapies under investigation. Thus, there may be a lower chance of a participant being randomized to the control arm (compared to a traditional study design with one control and one experimental therapy arm), which may reduce the reluctance of participation from the patient/parent perspective. From the trial conduct perspective, this leads to a smaller overall sample size (while achieving the same statistical power) which aligns with the ethical preference of enrolling as few pediatric patients as possible [16, 17]. Examples of platform trials in adult research include I-SPY2 in breast cancer [18, 19] and DIAN-TU in Alzheimer’s disease [20].

Although there are some successful examples of master protocols in adult studies [21], the use of master protocols in pediatric research is lagging behind, with the experience largely in the field of oncology [22]. As summarized by Khan and colleagues (2019), master protocols are primarily used in early phase trials focused on dose finding, safety, and an estimate of pharmacologic activity with the goal of carrying selected candidates forward in more definitive Phase III trials [23–33]. The experience outside of pediatric oncology is more limited. This systematic review was undertaken with the goal of updating the available information about and to stimulate the use of master protocols in pediatric drug development.

## Methods

This systematic review aimed to identify pediatric clinical trials that used master protocols and was based on searching two data sources: PubMed and ClinicalTrials.gov (Supplemental Table 1). The PubMed search was conducted on September 9th, 2022, and included articles published in the past 10 years that have terms ‘master protocol’ or ‘basket trial’ or ‘platform trial’ or ‘umbrella’ in the title or abstract. The articles were filtered to restrict to article type ‘Clinical Trial’, article language ‘English’, and species ‘Humans’. These articles’ abstract and title were screened by two authors (YL, HP) to include clinical trials only, and remove statistical methodology papers, cases series, and biomarker studies (Supplemental Fig. 1). Full texts of the remaining articles were further reviewed by the two authors to identify the studies that included pediatric participants (age < 18) in the trial.

The ClinicalTrials.gov search was conducted on September 30th, 2022, and included studies that were ‘First Posted’ by that date. The studies were filtered with terms ‘master protocol’ or ‘basket’ or ‘platform trial’ or ‘umbrella’ and inclusion of ‘Child’ in the study (Supplemental Table 1). The remaining studies were reviewed by two authors (JY, KB) to include interventional trials only and exclude observational studies and patient registries (Supplemental Fig. 1). Non-relevant studies were also removed, such as digital platform, behavioral and exercise studies. The study lists identified from PubMed and ClinicalTrials.gov were then cross compared to remove duplicates.

For the studies that have been included in this systematic review, data were extracted from the information on ClinicalTrials.gov, and the full text manuscript and their supplemental materials, if available, using a standardized form. General study information was extracted, including study type, study status, trial sponsor, therapeutic area, study start year (based on ‘Actual Study Start Date’ on ClinicalTrials.gov), clinical trial phase, estimated trial duration (estimated using the study start date and completion date or estimated completion date if the study was still ongoing), whether the drugs used in the trial were from single or multiple organizations, whether used for drug repurposing (considered ‘Yes’ if all drugs used in the trial had been approved for adult indication), and whether used to support registration. For study status, the categories include ‘registered/proposed’ (but not started yet), ‘ongoing’, ‘completed’, and ‘terminated’. For therapeutic area, oncology is a combined category, while other categories indicate a specific disease (e.g., COVID). This was chosen because oncology has been the primary area of application of master protocols, and it is of interest to understand if studies of any other diseases are starting to use master protocols.

Study characteristics that are specific to pediatric studies were also extracted, including the minimum and maximum age of the study participants, whether the study was part of a master protocol that also included adult patients, whether the pediatric drug dosing was the same as adults (considered ‘Yes’ if all the drugs included in the study used adult dosing), the type of pediatric dosing if any of the drugs were dosed differently from adults (e.g., weight-based, body surface area [BSA]-based), and whether the pediatric drug formulation was the same as adult. Based on the minimum age of the study participants, flags were created to indicate if the trial included participants that were younger than 16, 12, 6, or 2 years old, which aligns with ICH E11(R1) age categories [34]. Another flag was also created to indicate if the trial included only adolescent or older patients (age > = 12).

Lastly, important study design elements of the master protocol were collected, including number of test drug arms at the start-up of the trial, whether randomization was used, whether a concurrent control was implemented, the type of control arm (e.g., placebo only, standard of care [SOC], active [defined as having been approved by FDA]), type of randomization (adaptive or fixed) and randomization ratio. All the data were first extracted by one reviewer and then independently validated by another reviewer. Discrepancies between reviewers were discussed and resolved based on consensus.

As mentioned in the Introduction, we specifically define the study types as follows [3]:

- **Basket trial**, which is defined as a trial/study testing a single investigational drug or drug combination in multiple disease populations, defined by disease stage, histology, number of prior therapies, genetic or other biomarkers, or demographic characteristics (e.g., AMETHIST, TRAM-01).
- **Umbrella trial**, which is defined as a trial/study evaluating multiple investigational drugs administered as single drugs or as drug combinations in a single disease population, where all investigational drugs (or combinations) are enrolled at the same time and with no rolling arm option [1] (e.g., PRAM-2, Pegathor Lymphoma 205).
- **Platform trial**, which allows flexibility to add new treatment arms in the future (e.g., ALLTogether1; hybrid of different disease indications and different treatments or treatment combinations in the same trial, such as NCI-COG Pediatric MATCH).

As this review does not synthesize the results of included clinical trials and only considers their study designs, formal bias assessment is not applicable. All analyses are descriptive, and no hypothesis tests are conducted. Summary of design elements are reported as frequencies and proportions for categorical variables, and mean, standard deviation (SD), median, range, and interquartile range (IQR) for continuous variables. These statistics are calculated overall, by study type, by sponsor type, and by therapeutic indication (grouped into oncology, covid, and other). All analyses have been performed using R 4.2.1.

## Results

One hundred and eighty-seven studies from the PubMed search were screened and assessed for eligibility. After applying the inclusion and exclusion criteria, 11 pediatric master protocol studies were identified (Supplemental Fig. 1). In the ClinicalTrial.gov search, 399 studies were screened, and 38 studies were selected. Cross comparison between the two lists revealed that all 11 publications in PubMed appeared in the ClinicalTrial.gov list so the final analysis included 38 pediatric studies.

**Fig. 1:**
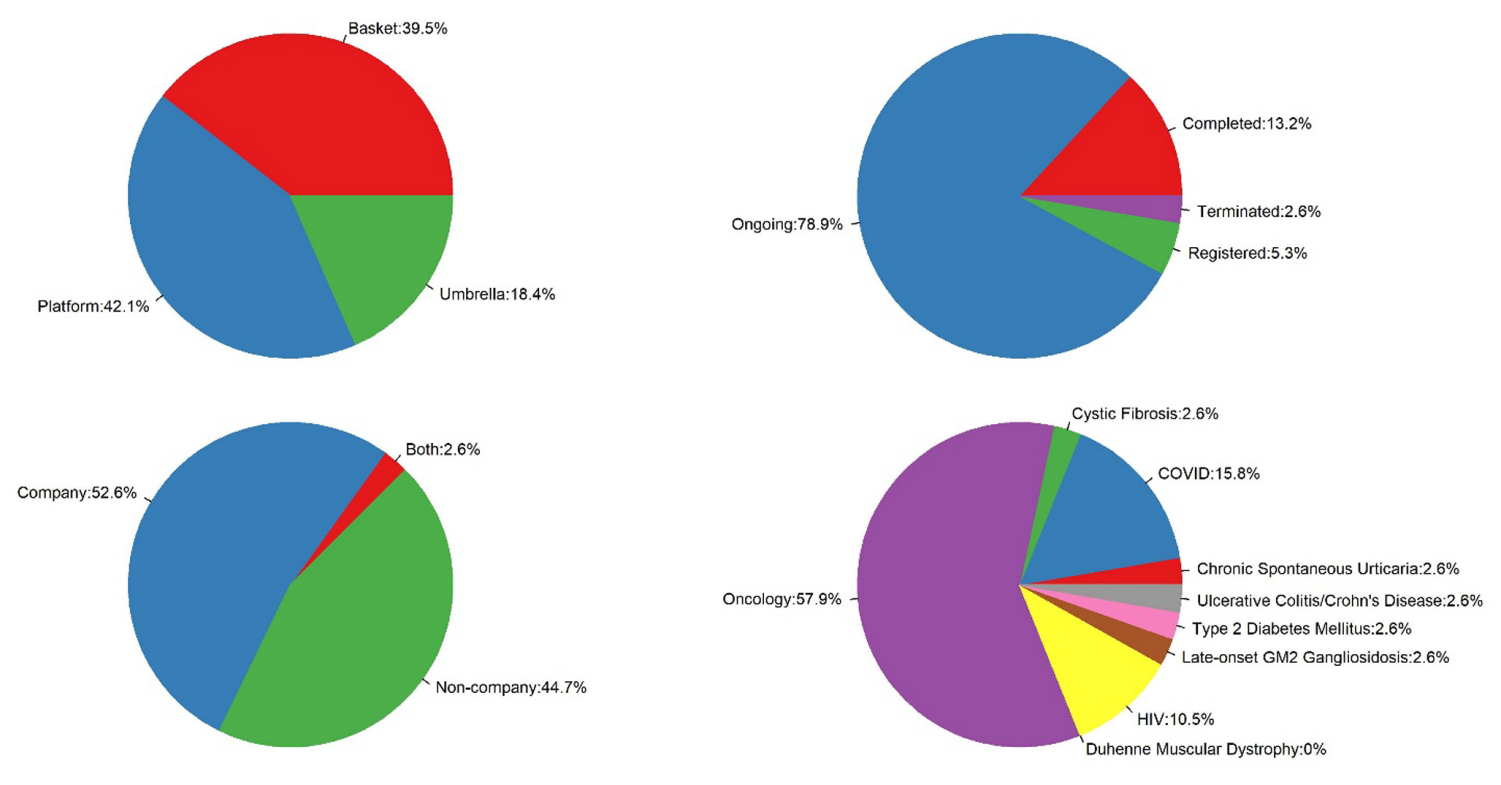
Summary of study type, study status, trial sponsor, and therapeutic area. *N* = 38

Figure 1 shows the distribution of study type, study status, trial sponsor, and therapeutic area. Among the 38 studies, 16 (42%) are platform trials, 15 (39%) are basket trials and 7 (18%) are umbrella studies. Most of the studies (79%) are still ongoing and 5 (13%) studies have been completed. The trial sponsor type is almost evenly distributed between company (52%) and non-company (45%), with one study sponsored by both a company and non-company. Oncology (58%) is the largest therapeutic area for the included studies, followed by infectious diseases treating COVID (18%) and HIV (8%). Among the 6 COVID studies, 3 are therapeutic and 3 are vaccine trials.

General study characteristics are summarized in Table 1, overall and by study type. The earliest pediatric trial that used a master protocol started in 1997, with a few (1 to 3) studies having started every year subsequently. The use of master protocols in pediatrics suddenly increased in 2020, with 10 studies starting in 2020 and another 10 studies starting in 2021 (the number in 2022 is underestimated because the search was conducted in September 2022). This rise was seen in both company and non-company sponsored trials (Supplemental Table 2), and mostly in the Oncology disease area (Supplemental Table 3). This increase in the past few years explains the observation that most studies are still ongoing. In terms of phases, the majority are early phase studies, with 60% Phase I or Phase I/II or Phase II studies. Most studies (61%) are not for drug repurposing and 47% trials use drugs from multiple organizations. Overall mean estimated trial duration for pediatric master protocols is 5.7 years, with a longer duration for platform trials (mean 7.3 years) compared to basket (mean 5.0 years) or umbrella trials (mean 3.7 years).

**Table 1:** General study characteristics, overall and by study type. One decimal only

Response	*N*	Overall, *N* = 38	Basket, *N* = 15	Platform, *N* = 16	Umbrella, *N* = 7
**Study status**	38				
Completed		5(13.2%)	1(6.7%)	2(12.5%)	2(28.6%)
Ongoing		30(79.0%)	11(73.3%)	14(87.5%)	5(71.4%)
Registered/proposed		2(5.3%)	2(13.3%)	0(0%)	0(0%)
Terminated		1(2.6%)	1(6.7%)	0(0%)	0(0%)
**Start year**	38				
1997		2(5.3%)	0(0%)	1(6.3%)	1(14.3%)
2001		1(2.6%)	0(0%)	1(6.3%)	0(0%)
2015		1(2.6%)	1(6.7%)	0(0%)	0(0%)
2016		2(5.2%)	1(6.7%)	1(6.3%)	0(0%)
2017		1(2.6%)	0(0%)	1(6.3%)	0(0%)
2018		3(7.9%)	2(13.3%)	0(0%)	1(14.3%)
2019		3(7.9%)	2(13.3%)	0(0%)	1(14.3%)
2020		10(26.3%)	5(33.3%)	4(25.0%)	1(14.3%)
2021		10(26.3%)	2(13.3%)	5(31.3%)	3(42.9%)
2022		4(10.5%)	1(6.7%)	3(18.8%)	0(0%)
2023		1(2.6%)	1(6.7%)	0(0%)	0(0%)
**Phases**	38				
Early phase (Phase I, I/II, II)		23(60.5%)	10(66.7%)	9(56.3%)	4(57.1%)
Other		15(39.5%)	5(33.3%)	7(43.8%)	3(42.9%)
**Drug repurposing/Off-label**	38				
No		23(60.5%)	9(60.0%)	9(56.3%)	5(71.4%)
Yes		15(39.5%)	6(40.0%)	7(43.8%)	2(28.6%)
**Results use to support registration**	38				
No		8(21.1%)	2(13.3%)	5(31.3%)	1(14.3%)
Unclear		22(57.9%)	8(53.3%)	9(56.3%)	5(71.4%)
Yes		8(21.1%)	5(33.3%)	2(12.5%)	1(14.3%)
**Trial sponsor**	38				
Both		1(2.6%)	1(6.7%)	0(0%)	0(0%)
Company		20(52.6%)	9(60.0%)	7(43.8%)	4(57.1%)
Non-company		17(44.7%)	5(33.3%)	9(56.3%)	3(42.9%)
**Drugs from same or different organizations**	38				
Multiple		18(47.4%)	4(26.7%)	10(62.5%)	4(57.1%)
Single		20(52.6%)	11(73.3%)	6(37.5%)	3(42.9%)
**Therapeutic area/indication**	38				
Chronic Spontaneous Urticaria		1(2.6%)	1(6.7%)	0(0%)	0(0%)
COVID		6(15.8%)	1(6.7%)	4(25.0%)	1(14.3%)
Covid Therapeutic		3	1	2	0
Covid Vaccine		3	0	2	1
Cystic Fibrosis		1(2.6%)	0(0%)	0(0%)	1(14.3%)
Duchenne Muscular Dystrophy		1(2.6%)	0(0%)	1(6.3%)	0(0%)
HIV		4(10.5%)	1(6.7%)	2(12.5%)	1(14.3%)
Late-onset GM2 Gangliosidosis		1(2.6%)	1(6.7%)	0(0%)	0(0%)
Oncology		22(57.9%)	11(73.3%)	8(50.0%)	3(42.9%)
Type 2 Diabetes Mellitus		1(2.6%)	0(0%)	0(0%)	1(14.3%)
Ulcerative Colitis/Crohn’s Disease		1(2.6%)	0(0%)	1(6.3%)	0(0%)
**Estimated trial duration, years**	38				
Mean (SD)		5.7 (3.8)	5.0 (2.1)	7.3 (5.1)	3.7 (1.7)
Median [IQR]		5.0 [4.0, 7.0]	5.00 [4.0, 6.5]	5.5 [3.8, 10.6]	4.0 [3.0, 4.5]
(Range)		(0.5, 20.0)	(2.0, 9.0)	(0.5, 20.0)	(1.0, 6.0)

**Fig. 2:**
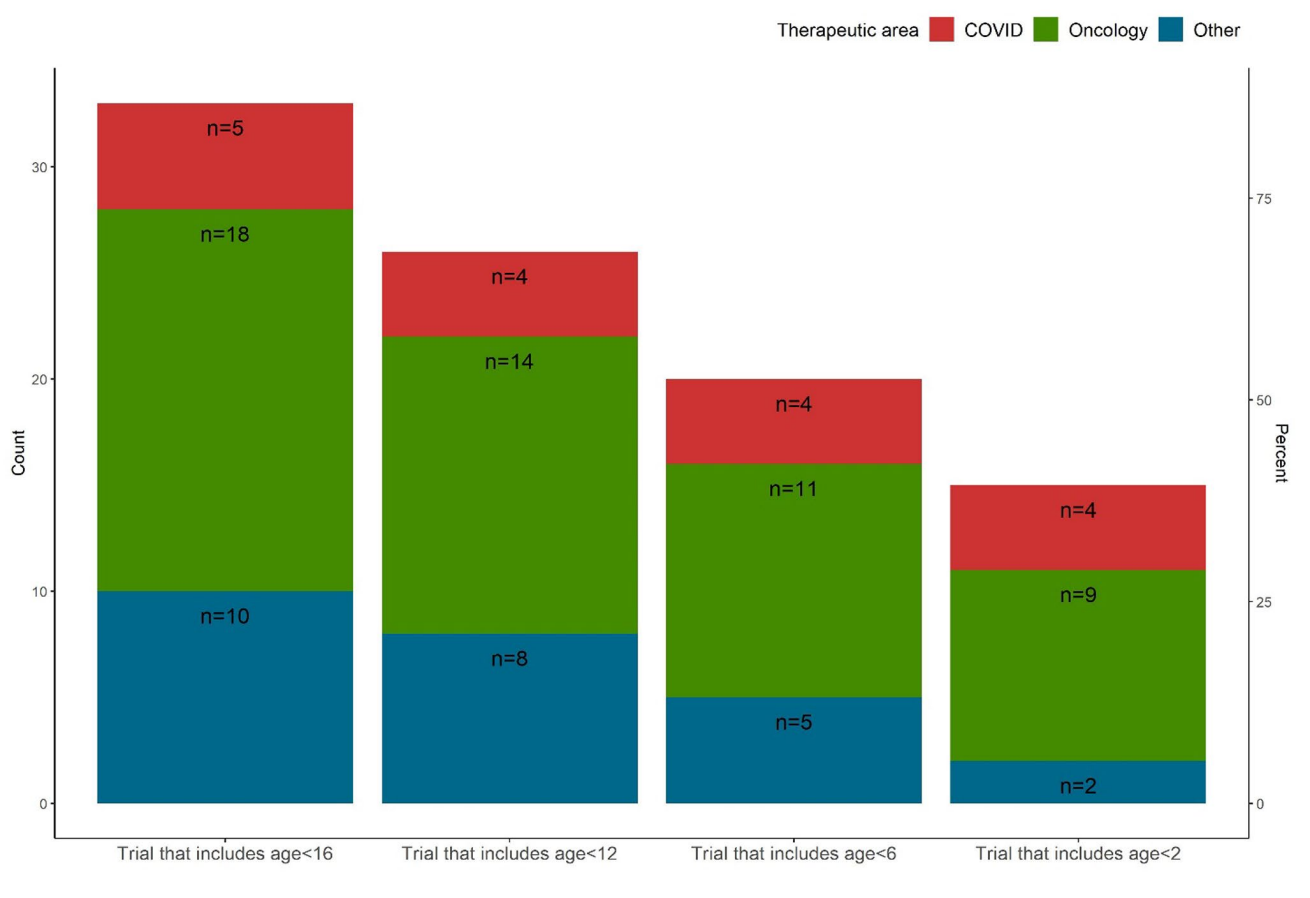
Number of trials that included patients younger than 16, 12, 6 or 2 years old, by therapeutic area

Pediatric specific characteristics are summarized in Table 2, overall and by study type. The minimum age of the study participants has a median of 2 with IQR 0.6 to 12. Among the 38 studies, 33 (87%) include participants younger than 16 years old, 26 (68%) include participants younger than 12 years old, 20 (53%) include participants younger than 6 years old, 15 (39%) include participants younger than 2 years old, and 12 (32%) include only adolescent or older participants. Interestingly, among the 6 COVID trials, 4 (3 therapeutic and 1 vaccine trials) include participants younger than 2 years old (Fig. 2). The minimum age of the study participants differs by study type, with a lower median for platform trials (1 year old) than basket (6 years old) or umbrella trials (12 years old). Twenty-six (68%) of the 38 pediatric studies are part of a master protocol that also included adult patients, and among them 12 studies (46%) include only adolescent or older patients. Twelve (32%) studies use the same dosing as adult, and 9 of these 12 studies include only adolescent or older patients. The most common type of pediatric dosing is weight-based dosing (46%), followed by BSA-based dosing (15%), and a combination of weight-based and BSA-based dosing (12%, e.g., weight-based dosing for one study drug and BSA-based dosing for another study drug).

**Table 2:** Pediatric specific characteristics, overall and by study type

	*N*	Overall, *N* = 38	Basket, *N* = 13	Platform, *N* = 16	Umbrella, *N* = 9
**Age Min, years**	38				
Mean (SD)		6.0 (6.1)	6.3 (5.9)	4.4 (6.1)	9.2 (5.8)
Median [IQR]		2.0 [0.6, 12.0]	6.0 [1.0, 11.0]	1.0 [0.0, 7.5]	12.0 [6.0, 12.0]
(Range)		(0.0, 16.0)	(0.0, 16.0)	(0.0, 16.0)	(0.3, 16.0)
**Trial includes age < 16**	38				
No		5(13.2%)	2(13.3%)	2(12.5%)	1(14.3%)
Yes		33(86.8%)	13(86.7%)	14(87.5%)	6(85.7%)
**Trial includes age < 12**	38				
No		12(31.6%)	4(26.7%)	4(25.0%)	4(57.1%)
Yes		26(68.4%)	11(73.3%)	12(75.0%)	3(42.9%)
**Trial includes age < 6**	38				
No		18(47.4%)	8(53.3%)	5(31.3%)	5(71.4%)
Yes		20(52.6%)	7(46.7%)	11(68.8%)	2(28.6%)
**Trial includes age < 2**	38				
No		23(60.5%)	10(66.7%)	7(43.8%)	6(85.7%)
Yes		15(39.5%)	5(33.3%)	9(56.3%)	1(14.3%)
**Trial with only adolescent or older**					
No		26(68.4%)	11(73.3%)	12(75.0%)	3(42.9%)
Yes		12(31.6%)	4(26.7%)	4(25.0%)	4(57.1%)
**Part of a master protocol that also included adult patients**	38				
No		12(31.6%)	4(26.7%)	6(37.5%)	2(28.6%)
Yes		26(68.4%)	11(73.3%)	10(62.5%)	5(71.4%)
Trial with only adolescent or older		12	4	4	4
Trial with younger patients		14	7	6	1
**Dosing same as adults**	38				
No		22(57.9%)	8(53.3%)	10(62.5%)	4(57.1%)
Unclear		4(10.5%)	1(6.7%)	3(18.8%)	0(0%)
Yes		12(31.6%)	6(40.0%)	3(18.8%)	3(42.9%)
Trial with only adolescent or older		9	4	3	2
Trial with younger patients		3	2	0	1
**Type of dosing, if not same as adults**	26				
Age-based		1(3.8%)	0(0%)	1(7.7%)	0(0%)
BSA-based		4(15.4%)	2(22.2%)	1(7.7%)	1(25.0%)
Unclear		5(19.2%)	2(22.2%)	3(23.1%)	0(0%)
Weight-based		12(46.2%)	4(44.4%)	6(46.2%)	2(50.0%)
Weight-based, age-based		1(3.8%)	0(0%)	1(7.7%)	0(0%)
Weight-based, BSA-based		3(11.5%)	1(11.1%)	1(7.7%)	1(25.0%)
**Formulation: Same as adults**	38				
No		9(23.7%)	1(6.7%)	7(43.8%)	1(14.3%)
Unclear		3(7.9%)	1(6.7%)	2(12.5%)	0(0%)
Yes		26(68.4%)	13(86.7%)	7(43.8%)	6(85.7%)

Table 3 summarizes certain elements related to the study design, overall and by study type. For basket trials, most of them (12 out of 15, 80%) have only 1 test drug arm (a combination therapy is considered as 1 test drug arm). For platform trials, only 13% (2 out of 16) have 1 test drug arm (with the plan to add more arms) and 44% (7 out 16) have 5 or more test drug arms at the start-up of the trial with potential to expand. Overall, 17 studies (45%) used randomization. Among the 17 studies that used randomization, the majority (13 out of 17, 76%) use a concurrent control, but the type of control arm varies, with 6 studies (35%) using a placebo control, 6 studies (35%) using an active control, and 3 studies (18%) using SOC. Of note, among the 6 trials that use a placebo control, only 1 study is an adolescent study. Among the studies that have provided information on the details of randomization, no study used adaptive randomization and most studies used 1:1 randomization ratio (11 out of 17, 65%).

**Table 3:** Elements of study design, overall and by study type

	*N*	Overall, *N* = 38	Basket, *N* = 13	Platform, *N* = 16	Umbrella, *N* = 9
**# of test drug arms (at start-up)**	38				
1		14(36.8%)	12(80.0%)	2(12.5%)	0(0%)
2		9(23.7%)	1(6.7%)	3(18.8%)	5(71.4%)
3		3(7.9%)	0(0%)	2(12.5%)	1(14.3%)
4		3(7.9%)	1(6.7%)	1(6.3%)	1(14.3%)
5 or more		8(21.1%)	1(6.7%)	7(43.8%)	0(0%)
Unclear		1(2.6%)	0(0%)	1(6.3%)	0(0%)
**Randomization, if used**	38				
No		21(55.3%)	11(73.3%)	8(50.0%)	2(28.6%)
Yes		17(44.7%)	4(26.7%)	8(50.0%)	5(71.4%)
Early phase (phase I, I/II, II)		5	0	3	2
Other phase		12	4	5	3
**Control, concurrent or nonconcurrent, among those with randomization**	17				
Concurrent		13(81.3%)	4(100.0%)	5(62.5%)	4(80.0%)
Concurrent and non-concurrent		1(6.3%)	0(0%)	1(12.5%)	0(0%)
No control arm		1(6.3%)	0(0%)	1(12.5%)	1(20.0%)
unclear		1(6.3%)	0(0%)	1(12.5%)	0(0%)
**Type of control arm, among those with randomization**	17				
No control arm		1(5.9%)	0(0%)	0(0%)	1(20.0%)
Active		6(35.3%)	1(25.0%)	4(50.0%)	1(20.0%)
SOC		3(17.7%)	0(0%)	3(37.5%)	0(0%)
Randomized withdrawal		1(5.9%)	0(0%)	0(0%)	1(20.0%)
Placebo only		6(35.3%)	3(75.0%)	1(12.5%)	2(40.0%)
Trial with only adolescent or older		1	0	0	1
Trial with younger patients		5	3	1	1
**Randomization ratio, fixed or adaptive, among those with randomization**	17				
Fixed		14(82.4%)	2(50.0%)	7(87.5%)	5(100.0%)
Unclear		3(17.7%)	2(50.0%)	1(12.5%)	0(0%)
**Randomization ratio, among those with randomization**	17				
1:1		11(64.7%)	2(50.0%)	6(75.0%)	3(60.0%)
Unclear		5(29.4%)	2(50.0%)	1(12.5%)	2(40.0%)
Unequal (2:1)		1(5.9%)	0(0%)	1(12.5%)	0(0%)

All the study characteristics are also summarized by sponsor type (Supplemental Table 2) and by therapeutic area (Supplemental Table 3). A few characteristics vary by sponsor type; for example, trials sponsored by a company are more likely to include drugs from a single (rather than more than one) organization and are more likely to use a placebo (rather than an active) control arm, compared to the trials sponsored by a non-company. For comparison by therapeutic area, oncology trials are more likely in early phases, more likely to have one (rather than more than one) test drug arm, and less likely to use randomization, compared to trials for other indications.

## Discussion

This systematic review showed that master protocol studies are being used in pediatrics, with platform and basket trials more common than umbrella trials. As expected, most experience with the use of master protocols is in oncology, with early phase studies more common than late phase. There has been a rise in the number of pediatric master protocols starting in 2020, again largely in oncology. It is speculated that the rise is a result of increasing awareness of master protocols. In particular, oncology is an opportune area to apply pediatric master protocols, given the previous success with adult oncology master protocols [18, 19, 35–38]. The application of master protocols has also emerged in COVID-19 therapeutic and vaccine trials, and FDA has published specific guidelines for COVID-19 master protocols [39], in addition to guidelines for oncology [3]. The COVID-19 pandemic has led to many examples of advances in clinical trial designs such as master protocols to deliver critical data to inform patient management [40–44]. However, the FDA assessment has been that the vast number of these global clinical trials were not randomized or adequately powered. As a result, the majority of these innovative designs were unable to generate actionable data of sufficient quality [45].

Our results show that many of the pediatric studies are in the same master protocols as adults, and they include both adolescent studies and pediatric studies with younger age groups. This suggests that the master protocol studies need not be solely pediatric unless perhaps when the indication itself is primarily for a pediatric group (e.g., rare disorders). Conceptually, the relatively easy way to incorporate pediatric participants in every therapeutic area, especially adolescents, may be to broaden the inclusion criteria in the same adult protocol and adjust the treatment and/or outcome for the pediatric age groups. This approach could potentially leverage adult information for pediatrics and allow for generalization across entire study populations [46]. Additionally, we also found that about one third of the pediatric master protocols use the same dosing as adults (a flat dose), and the majority of these studies are adolescent trials.

As expected, and confirmed by our review, platform and umbrella trials include multiple test drug arms, or plan to add more arms if starting with only one arm. Basket trials mostly have one test drug arm, although occasionally include multiple arms. The involvement of multiple drugs requires access to these drugs, either all from a single organization leading the trial or a collaboration among multiple organizations agreeing to participate in the trial. Not surprisingly, we found that trials sponsored by a company are more likely to include drugs from a single rather than multiple organizations, compared to the trials sponsored by a non-company.

For pediatric master protocols with randomization, no study in our review has been identified as using adaptive randomization. This may be due to the reluctance of adding another layer of complexity to an already complicated master protocol study design. When evaluating the type of control arms in randomized studies, we observed that the use of SOC is infrequent, although this was recommended by FDA guidance [3]. Interestingly, a few studies use placebo as a control arm, and most of these studies are not adolescent only trials. In general, there have been concerns as to whether a placebo arm is feasible in pediatric studies [47], but our results suggest that pediatric master protocols are able to recruit patients into placebo-controlled studies.

Our study has a few limitations. First, our review included studies that had inclusion/exclusion criteria allowing adolescent as well as pediatric participants. The studies cover diseases that are solely diagnosed in pediatric patients, as well as diseases are in both adult and pediatric patients. Further work is needed to understand the reasons why some master protocols did not include younger age groups. Second, we were unable to collect key drivers as to why an organization adopted a master protocol design in the first place rather than conduct a more traditional clinical trial. One example is whether the master protocol study results would be used to support registration, which is difficult to determine using public information.

The use of master protocols in pediatric research could potentially improve trial efficiency and reduce the costs of drug development; for example, by including pediatric patients in adult master protocols. Another opportunity would be the use of a master protocol by a single sponsor, for example, when multiple products in the pipeline are being developed for the same indication. What is aspirational and would require more efforts is building collaborations across several sponsors, including pediatric (or rare disease) registries to facilitate master protocols. The development of cross-sponsor collaborations and a shared trial infrastructure may impose logistical difficulties with coordinating and communicating across multiple companies and raise issue about ownership of the trial. However, once agreement is reached and the infrastructure established, it will make evaluation of new therapies easier and faster and provide assurance that necessary and sufficient data in pediatrics will be collected. Hopefully, there will be a paradigm shift from a single entity conducting a pediatric trial to multiple entities collaborating together. EU-PEARL (EU Patient-cEntric clinnicAl tRial pLatforms) serves as a good example of partnership [48], which offers a useful model for pediatric platform trials.

## Conclusion

Master protocols offer the potential for improved pediatric studies by creating efficiencies in clinical operations and possibly using shared control groups. Our review shows that master protocols are starting to be adopted in pediatric clinical research, but on a small scale that could be substantially expanded. Work is required to further understand the barriers in implementing pediatric master protocols, from setting up shared infrastructure to interpreting study findings. The ultimate goal would be to reduce the delay in pediatric marketing approvals to allow timely access to safe and effective therapies for children.

### Electronic Supplementary Material

Below is the link to the electronic supplementary material.

Supplementary Material 1

## Declarations

### Disclaimer

Dr. Sun is an employee of the FDA. This manuscript reflects the views of the author and should not be constructed to represent FDA’s views or policies.
