# Supplementary material for: Unlocking the Potential: A Systematic Review of Master Protocol in Pediatrics
Source: Ther Innov Regul Sci. 2024 Apr 23;58(4):634–44. doi: 10.1007/s43441-024-00656-z (PMC11169036; doi:10.1007/s43441-024-00656-z)
Supplement: Supplementary file 1 — Supplementary Material 1 [file 43441_2024_656_MOESM1_ESM.pdf]

Supplemental materials for the manuscript "Unlocking the Potential: A systematic review of Master Protocol in Pediatrics"

Supplemental Figure 1: Search process for the systematic review. *See Supplemental Table 1 for specific search criteria.

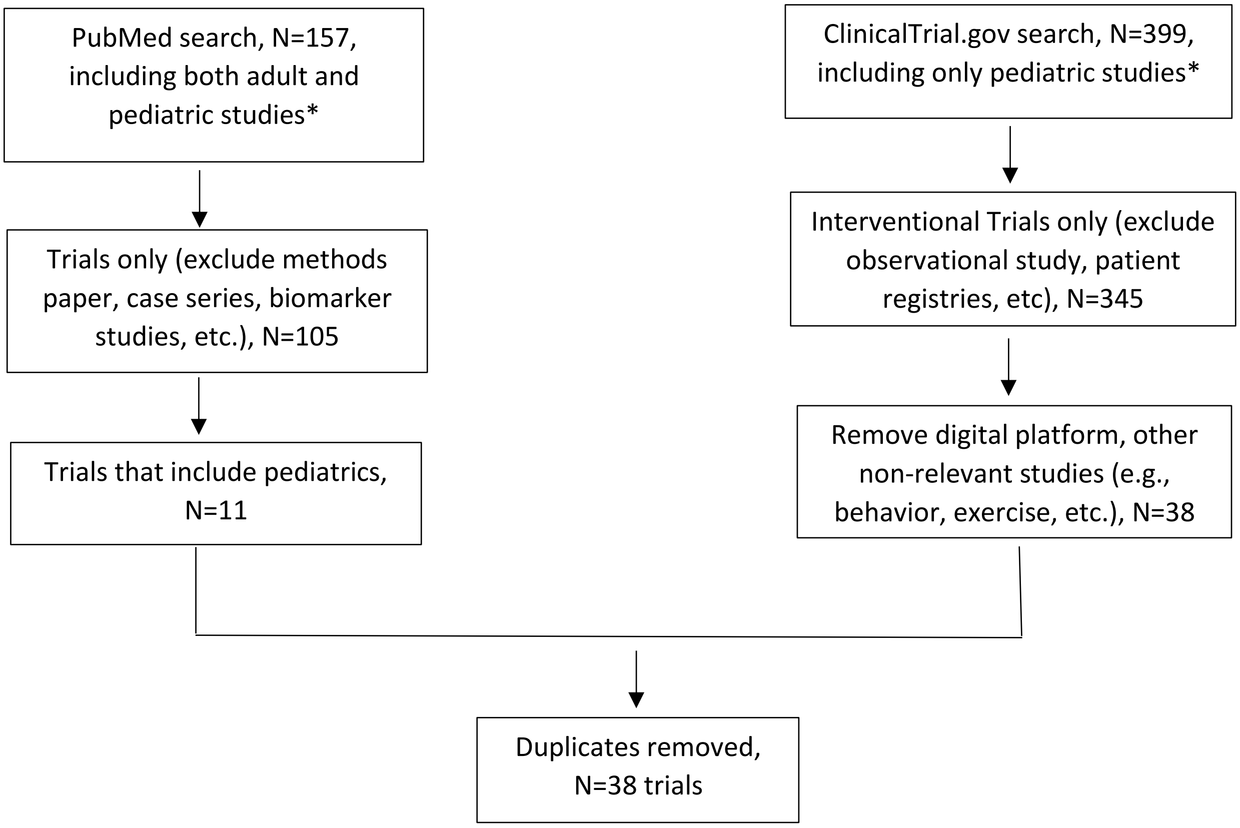

Supplemental Table 1: Specific search criteria

Source	Search Criteria
PubMed	Search conducted on Sept 9th 2022 at 11:37am EST, using the searching criteria: (((master protocol[Title/Abstract]) OR (basket trial[Title/Abstract])) OR (platform trial[Title/Abstract])) OR (umbrella[Title/Abstract]) Then filter to 'Clinical Trials', publication date '10 years', language 'English' (no change) and 'Human' (no change).
ClinicalTrials.gov	Search conducted on Sep. 30, 2022. Study First Posted by Sep. 30, 2022, Other terms: (((master protocol) OR (basket) OR (platform trial) OR (umbrella) AND **filter Child (birth-17)**

Supplemental Table 2: Study characteristics by trial sponsor. N=37. One study that is sponsored by both company and non-company is removed in this analysis.

	N	**Company**, N = 20	**Non-company**, N = 17
**Study status**	37		
Completed		2(10.0~~0~~%)	3(17.7~~65~~%)
Ongoing		15(75.0~~0~~%)	14(82.4~~35~~%)
Registered/proposed		2(10.0~~0~~%)	0(0%)
Terminated		1(5.0~~00~~%)	0(0%)
**Start year**	37		
1997		0(0%)	2(11.8~~76~~%)
2001		1(5.0~~00~~%)	0(0%)
2015		1(5.0~~00~~%)	0(0%)
2016		1(5.0~~00~~%)	1(5.9~~882~~%)
2017		0(0%)	1(5.~~882~~9%)
2018		1(5.0~~00~~%)	2(11.~~76~~8%)
2019		3(15.0~~0~~%)	0(0%)
2020		4(20.0~~0~~%)	5(29.4~~1~~%)
2021		5(25.0~~0~~%)	5(29.4~~1~~%)
2022		3(15.0~~0~~%)	1(5.~~882~~9%)
2023		1(5.0~~00~~%)	0(0%)
**Drug repurposing~~e~~/Off-label**	37		
No		13(65.0~~0~~%)	9(52.9~~4~~%)
Yes		7(35.0~~0~~%)	8(47.1~~06~~%)
**Phases**	37		
Early phase (I, I/II, II)		10(50.0~~0~~%)	12(70.6~~59~~%)
Other		10(50.0~~0~~%)	5(29.4~~1~~%)
**Results use to support registration**	37		
No		2(10.0~~0~~%)	5(29.4~~1~~%)
Unclear		11(55.0~~0~~%)	11(64.7~~1~~%)
Yes		7(35.0~~0~~%)	1(5.9~~882~~%)
**Drugs from same or different organizations**	37		
Multiple		5(25.0~~0~~%)	13(76.5~~47~~%)
Single		15(75.0~~0~~%)	4(23.5~~3~~%)
**Therapeutic area/indication**	37		
Chronic Spontaneous Urticaria		1(5.0~~00~~%)	0(0%)
COVID		4(20.0~~0~~%)	2(11.8~~76~~%)
Cystic Fibrosis		0(0%)	1(5.~~882~~9%)
Duchenne Muscular Dystrophy		1(5.0~~00~~%)	0(0%)
HIV		1(5.0~~00~~%)	3(17.~~65~~7%)
Late-onset GM2 Gangliosidosis		1(5.0~~00~~%)	0(0%)
Oncology		10(50.0~~0~~%)	11(64.7~~1~~%)
Type 2 Diabetes Mellitus		1(5.0~~00~~%)	0(0%)
Ulcerative Colitis/Crohn's Disease		1(5.0~~00~~%)	0(0%)

	N	**Company**, N = 20	**Non-company**, N = 17
**Estimated trial duration, years**	37		
Mean (SD)		4.~~57~~ 6 (2.~~6~~7)	7.~~18~~ 2 (4.6~~1~~)
Median [IQR]		4.0~~0~~ [3.2~~2~~, 5.0~~0~~]	6.0~~0~~ [4.0~~0~~, 10.0~~0~~]
(Range)		(0.5~~0~~, 12.0~~0~~)	(1.6~~58~~, 20.0~~0~~)
**Dosing: Same as adults**	37		
No		11(55.0~~0~~%)	10(58.8~~2~~%)
Unclear		3(15.0~~0~~%)	1(5.9~~882~~%)
Yes		6(30.0~~0~~%)	6(35.3~~29~~%)
**Dosing: if not same as adults, type of dosing**	25		
Age-based		1(7.1~~43~~%)	0(0%)
BSA-based		3(21.4~~3~~%)	1(9.~~09~~1%)
Unclear		4(28.~~57~~6%)	1(9.~~09~~1%)
Weight-based		6(42.~~86~~9%)	6(54.6~~55~~%)
Weight-based, age-based		0(0%)	1(9.~~09~~1%)
Weight-based, BSA-based		0(0%)	2(18.2~~18~~%)
**Formulation: Same as adults**	37		
No		2(10.0~~0~~%)	7(41.~~18~~2%)
Unclear		2(10.0~~0~~%)	1(5.~~882~~9%)
Yes		16(80.0~~0~~%)	9(52.9~~4~~%)
**Age Min**	37		
Mean (SD)		6.6~~2~~ (5.7~~2~~)	5.3~~1~~ (6.~~75~~8)
Median [IQR]		6.0~~0~~ [1.0~~0~~, 12.0~~0~~]	1.0~~0~~ [0.~~08~~1, 12.0~~0~~]
(Range)		(0.0~~0~~, 16.0~~0~~)	(0.0~~0~~, 16.0~~0~~)
**Trial includes age<16**	37		
No		2(10.0~~0~~%)	3(17.~~65~~7%)
Yes		18(90.0~~0~~%)	14(82.~~35~~4%)
**Trial includes age<12**	37		
No		6(30.0~~0~~%)	6(35.~~29~~3%)
Yes		14(70.0~~0~~%)	11(64.7~~1~~%)
**Trial includes age<6**	37		
No		11(55.0~~0~~%)	6(35.~~29~~3%)
Yes		9(45.0~~0~~%)	11(64.7~~1~~%)
**Trial includes age<2**	37		
No		14(70.0~~0~~%)	8(47.~~06~~1%)
Yes		6(30.0~~0~~%)	9(52.9~~4~~%)
**Trial with only adolescent or older**	37		
No		14(70.0~~0~~%)	11(64.7~~1~~%)
Yes		6(30.0~~0~~%)	6(35.~~29~~3%)
**Part of a ~~In same adult~~ master protocol that also included adult patients ~~or not~~**	37		
No		5(25.0~~0~~%)	7(41.~~18~~2%)
Yes		15(75.0~~0~~%)	10(58.8~~2~~%)

	N	**Company**, N = 20	**Non-company**, N = 17
**# of test drug arms (at start-up)**	37		
1		9(45.0~~0~~%)	6(35.3~~29~~%)
2		5(25.0~~0~~%)	2(11.~~76~~8%)
3		1(5.0~~00~~%)	2(11.~~76~~8%)
4		1(5.0~~00~~%)	2(11.~~76~~8%)
5 or more		4(20.0~~0~~%)	4(23.5~~3~~%)
Unclear		0(0%)	1(5.9~~882~~%)
**Randomization, if used**	37		
No		11(55.0~~0~~%)	9(52.9~~4~~%)
Yes		9(45.0~~0~~%)	8(47.1~~06~~%)
**Control, concurrent or nonconcurrent, among those with randomization**	17		
Concurrent		8(88.~~8~~9%)	5(62.5~~0~~%)
Concurrent and non-concurrent		0(0%)	1(12.5~~0~~%)
No control arm		1(11.1~~1~~%)	1(12.5~~0~~%)
unclear		0(0%)	1(12.5~~0~~%)
**Type of control arm, among those with randomization**	17		
Active		2(22.2~~2~~%)	4(50.0~~0~~%)
No control arm		0(0%)	1(12.5~~0~~%)
Placebo only		6(66.~~6~~7%)	0(0%)
Randomized withdrawal		0(0%)	1(12.5~~0~~%)
SOC		1(11.1~~1~~%)	2(25.0~~0~~%)
**Randomization ratio, fixed or adaptive, among those with randomization**	17		
Fixed		6(66.~~6~~7%)	8(100.0~~0~~%)
Unclear		3(33.3~~3~~%)	0(0%)
**Randomization ratio, among those with randomization**	17		
1:1		4(44.4~~4~~%)	7(87.5~~0~~%)
Unclear		4(44.4~~4~~%)	1(12.5~~0~~%)
Unequal (2:1)		1(11.1~~1~~%)	0(0%)

Supplemental Table 3: Study characteristics by therapeutic area.

	N	**COVID**, N = 6	**Oncology**, N = 22	**Other**, N = 10
**Study status**	38			
Completed		0(0%)	1(4.6545%)	4(40.00%)
Ongoing		5(83.33%)	19(86.436%)	6(60.00%)
Registered/proposed		0(0%)	2(9.091%)	0(0%)
Terminated		1(16.67%)	0(0%)	0(0%)
**Start year**	38			
1997		0(0%)	0(0%)	2(20.00%)
2001		0(0%)	0(0%)	1(10.00%)
2015		0(0%)	1(4.6545%)	0(0%)
2016		0(0%)	0(0%)	2(20.00%)
2017		0(0%)	1(4.5456%)	0(0%)
2018		0(0%)	2(9.091%)	1(10.00%)
2019		0(0%)	2(9.091%)	1(10.00%)
2020		2(33.33%)	6(27.327%)	2(20.00%)
2021		2(33.33%)	7(31.82%)	1(10.00%)
2022		2(33.33%)	2(9.091%)	0(0%)
2023		0(0%)	1(4.6545%)	0(0%)
**Drug repurposinge/Off-label**	38			
No		4(66.67%)	12(54.655%)	7(70.00%)
Yes		2(33.33%)	10(45.45%)	3(30.00%)
**Phases**	38			
Early phase (I, I/II, II)		0(0%)	20(90.91%)	3(30.00%)
Other		6(100.00%)	2(9.091%)	7(70.00%)
**Results use to support registration**	38			
No		0(0%)	6(27.273%)	2(20.00%)
Unclear		3(50.00%)	15(68.182%)	4(40.00%)
Yes		3(50.00%)	1(4.5456%)	4(40.00%)
**Trial sponsor**	38			
Both		0(0%)	1(4.5456%)	0(0%)
Company		4(66.67%)	10(45.45%)	6(60.00%)
Non-company		2(33.33%)	11(50.00%)	4(40.00%)
**Drugs from same or different organizations**	38			
Multiple		2(33.33%)	10(45.45%)	6(60.00%)
Single		4(66.67%)	12(54.655%)	4(40.00%)
**Estimated trial duration, years**	38			
Mean (SD)		3.35 (4.33)	6.74 (4.107)	4.93 (2.00)
Median [IQR]		1.879 [1.215, 2.875]	5.00 [4.00, 8.67]	4.50 [4.00, 5.875]
(Range)		(0.50, 12.00)	(2.00, 20.00)	(2.00, 9.00)
**Dosing: Same as adults**	38			
No		4(66.67%)	11(50.00%)	7(70.00%)

	N	**COVID**, N = 6	**Oncology**, N = 22	**Other**, N = 10
Unclear		0(0%)	3(13.64%)	1(10.00%)
Yes		2(33.33%)	8(36.436%)	2(20.00%)
**Dosing: if not same as adults, type of dosing**	26			
Age-based		1(25.00%)	0(0%)	0(0%)
BSA-based		0(0%)	4(28.657%)	0(0%)
Unclear		0(0%)	3(21.43%)	2(25.00%)
Weight-based		3(75.00%)	5(35.71%)	4(50.00%)
Weight-based, age-based		0(0%)	1(7.143%)	0(0%)
Weight-based, BSA-based		0(0%)	1(7.143%)	2(25.00%)
**Formulation: Same as adults**	38			
No		2(33.33%)	5(22.73%)	2(20.00%)
Unclear		0(0%)	2(9.091%)	1(10.00%)
Yes		4(66.67%)	15(68.218%)	7(70.00%)
**Age Min**	38			
Mean (SD)		4.89 (7.217)	6.64 (6.547)	5.34 (4.985)
Median [IQR]		0.768 [0.13, 9.21]	4.00 [1.00, 12.00]	4.00 [2.00, 9.00]
(Range)		(0.00, 16.00)	(0.00, 16.00)	(0.108, 13.00)
**Trial includes age<16**	38			
No		1(16.67%)	4(18.182%)	0(0%)
Yes		5(83.33%)	18(81.82%)	10(100.00%)
**Trial includes age<12**	38			
No		2(33.33%)	8(36.364%)	2(20.00%)
Yes		4(66.67%)	14(63.64%)	8(80.00%)
**Trial includes age<6**	38			
No		2(33.33%)	11(50.00%)	5(50.00%)
Yes		4(66.67%)	11(50.00%)	5(50.00%)
**Trial includes age<2**	38			
No		2(33.33%)	13(59.109%)	8(80.00%)
Yes		4(66.67%)	9(40.91%)	2(20.00%)
**Trial with only adolescent or older**				
No		4(66.67%)	14(63.64%)	8(80.00%)
Yes		2(33.33%)	8(36.436%)	2(20.00%)
**Part of a ~~In same adult~~ master protocol that also included adult patients ~~or not~~**	38			
No		2(33.33%)	5(22.73%)	5(50.00%)
Yes		4(66.67%)	17(77.327%)	5(50.00%)
**# of test drug arms (at start-up)**	38			
1		2(33.33%)	13(59.109%)	1(10.00%)
2		0(0%)	3(13.64%)	4(40.00%)
3		1(16.67%)	1(4.6545%)	1(10.00%)

	N	**COVID**, N = 6	**Oncology**, N = 22	**Other**, N = 10
4		1(16.~~6~~7%)	0(0%)	2(20.0~~0~~%)
5 or more		2(33.~~3~~3%)	4(18.2~~18~~%)	2(20.0~~0~~%)
Unclear		0(0%)	1(4.6~~545~~%)	0(0%)
**Randomization, if used**	38			
No		1(16.~~6~~7%)	19(86.4~~36~~%)	1(10.0~~0~~%)
Yes		5(83.3~~3~~%)	3(13.6~~4~~%)	9(90.0~~0~~%)
**Control, concurrent or nonconcurrent, among those with randomization**	17			
Concurrent		4(80.0~~0~~%)	2(66.~~6~~7%)	7(77.~~7~~8%)
Concurrent and non-concurrent		0(0%)	0(0%)	1(11.1~~1~~%)
No control arm		0(0%)	1(33.3~~3~~%)	1(11.1~~1~~%)
unclear		1(20.0~~0~~%)	0(0%)	0(0%)
**Type of control arm, among those with randomization**	17			
Active		2(40.0~~0~~%)	0(0%)	4(44.4~~4~~%)
No Control arm		0(0%)	1(33.3~~3~~%)	0(0%)
Placebo only		2(40.0~~0~~%)	0(0%)	4(44.4~~4~~%)
Randomized withdrawal		0(0%)	0(0%)	1(11.1~~1~~%)
SOC		1(20.0~~0~~%)	2(66.~~6~~7%)	0(0%)
**Randomization ratio, fixed or adaptive, among those with randomization**	17			
Fixed		5(100.0~~0~~%)	3(100.0~~0~~%)	6(66.~~6~~7%)
Unclear		0(0%)	0(0%)	3(33.~~3~~3%)
**Randomization ratio, among those with randomization**	17			
1:1		4(80.0~~0~~%)	2(66.~~6~~7%)	5(55.~~5~~6%)
Unclear		1(20.0~~0~~%)	1(33.3~~3~~%)	3(33.3~~3~~%)
Unequal (2:1)		0(0%)	0(0%)	1(11.1~~1~~%)
